# Exploring the association between schizophrenia and multiple sclerosis: a case report

**DOI:** 10.1192/j.eurpsy.2025.769

**Published:** 2025-08-26

**Authors:** T. Pessoa, B. Silva, Â. Pinto, Â. Venâncio

**Affiliations:** 1 Psychiatry, ULSGE, Vila Nova de Gaia, Portugal

## Abstract

**Introduction:**

Schizophrenia (SCZ) is a chronic psychiatric disorder affecting cognition, perception, and behavior, while multiple sclerosis (MS) is a neurological disorder impacting the central nervous system. Despite unclear etiologies, both share commonalities such as immune-inflammatory pathways, neurocognitive impairment, and sleep disturbances (Misiak et al., 2023). Psychotic symptoms in MS typically follow neurological onset. This case report presents a patient diagnosed with SCZ a decade before developing neurological symptoms consistent with MS. His psychotic relapses coinciding with MS onset suggest potential interactions between the two disorders, raising questions about their connection.

**Objectives:**

To investigate the potential link between SCZ and MS through a case report of a patient with SCZ who developed MS a decade later. This study aims to explore the relationship between MS onset and psychotic relapses, focusing on shared neuroinflammatory mechanisms and their implications for co-occurring SCZ and MS.

**Methods:**

We present a case report and a non-systematic review on the subject.

**Results:**

Male patient, 33 years old, diagnosed with SCZ at the age of 23. No previous hospitalisations. No other comorbidities. Stabilised with aripiprazole 10mg for several years. In April 2023, the patient presented to the emergency department with binocular oblique diplopia on levoversion, which had progressed for 4 days due to ophthalmoparesis. Initial diagnostic tests, including an analytical study and a CT scan, showed no abnormalities. He was referred to neurology for further investigation. He underwent an electromyography with repetitive nerve stimulation, which was normal, and an MRI-CE which revealed multiple focal hyperintense areas in T2 and T2-FLAIR in the white matter suggestive of inflammatory demyelinating lesions, consistent with MS. In December 2023, the patient was hospitalized due to psychotic decompensation. He was discharged in January 2024 stabilised with aripiprazole 30mg. However, in March 2024 he was re-hospitalized with another psychotic decompensation attributed to non-compliance with medication. Aripiprazole was reintroduced and transitioned to a long-acting injectable formulation, and the patient was discharged.

**Conclusions:**

This case highlights important aspects of the relationship between SCZ and MS. The patient’s decade-long stable psychiatric history before MS onset suggests that neuroinflammation from MS may have triggered or worsened psychotic symptoms. The neuroinflammatory processes and immune-mediated mechanisms, common to both SCZ and MS, potentially explain their co-occurrence. The timeline of neurological symptoms preceding psychiatric relapse strengthens this connection. Further research is needed to clarify the shared pathophysiology between these disorders and guide effective treatments for individuals affected by both conditions.

**Disclosure of Interest:**

None Declared

